# Response to Letter to the Editor: Do Age-related Differences in the Incidence of Mumps Deafness Reflect a True Difference or a Misclassification of Mumps Deafness?

**DOI:** 10.2188/jea.JE20210098

**Published:** 2022-01-05

**Authors:** Akira Takagi, Satoko Ohfuji, Takashi Nakano, Hideaki Kumihashi, Munehide Kano, Toshihiro Tanaka

**Affiliations:** 1 Department of Otorhinolaryngology, Head and Neck Surgery, Shizuoka General Hospital, Shizuoka, Japan; 2 Department of Public Health, Osaka City University Graduate School of Medicine and Research Center for Infectious Disease Sciences, Osaka City University Graduate School of Medicine, Osaka, Japan; 3 Department of Pediatrics, Kawasaki Medical School, Okayama, Japan; 4 Global Vaccine Business Unit, Takeda Pharmaceutical Company Limited, Tokyo, Japan; 5 Department of Pediatrics, Shizuoka Kosei Hospital, Shizuoka, Japan

We thank the editor for the opportunity to respond to the comments and concerns raised by Dr Takashi Fujiwara and Dr Yohei Maeda^1^ with regard to our paper.^2^

**Figure 1.**
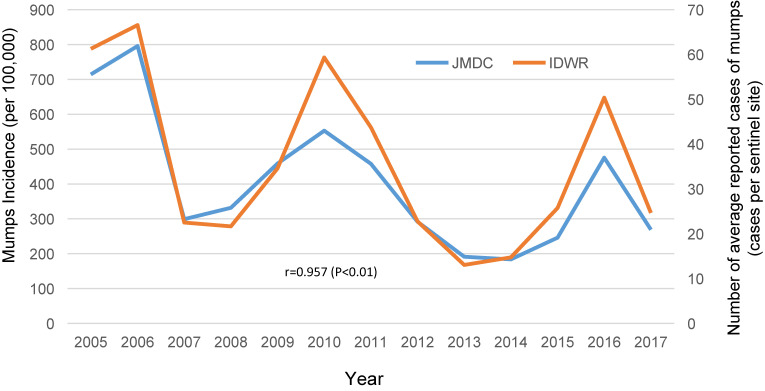
Mumps incidence (/100,000 persons: JMDC) and number of average reported cases of mumps per sentinel site (IDWR^3^)

We agree that validation is important in database studies. The Japan Medical Data Center database only provides anonymously processed information, making it difficult to link to the original medical records. Accordingly, we could not trace information back to the original medical records to confirm the appropriateness of the assigned disease code. Admittedly, this is one of the limitations of our study. However, an additional analysis of the comparison between the annual change in the number of diagnosed cases of mumps over the years in our study and data from the existing National Epidemiological Surveillance of Infectious Diseases,^3^ shows similarities between the two (Figure 1), and the correlation coefficient is high. In addition, as described in the text, our results were similar to those of a prospective study (Hashimoto et al) of mumps deafness in a population aged 20 years or younger.^4^

As you are aware, mumps usually occurs during childhood. In the study presented by Nagai et al,^5^ the number of reported cases of mumps was estimated based on sentinel site information in pediatric practice. Therefore, in terms of age, adults who did not see a pediatrician were not included in the denominator. Because the incidence of mumps differs between adults and children,^6^ application of the results reported by Nagai et al^5^ to the full population may result in an overestimation of the disease. In our study over 13 years from 2005 to 2017, the number of subjects included in the analysis of mumps (all ages) was 5,209,660 (see Figure 1 in the original text), and the number of subjects with mumps was 62,551 (see Table 1 in the original text).^2^

In our study, sudden deafness was included under acute sensorineural deafness, although this inclusion was presented as supplementary data and may therefore have escaped notice (see eTable 1 in the original publication).^2^ For the purposes of analysis, we included sudden deafness under acute sensorineural deafness, but included only cases of deafness where a mumps antibody test had been performed; therefore, it seems unlikely that sudden deafness was accidentally included under mumps deafness. We nevertheless acknowledge that this is another limitation of our study. The incidence of mumps deafness in our study was based on the number of patients with mumps. Since the number of adult patients with mumps peaks between 26 and 35 years of age and then decreases with age, the number of patients with mumps deafness also decreases with age, whereas the incidence of mumps deafness, which is based on the number of patients with mumps, tends to increase as the population (denominator) decreases. In other words, older people are less likely to have mumps, but if they do, they are more likely to become deaf, compared with children. According to a paper written by Fujiwara et al, on the other hand, the number of cases and incidence of sudden deafness increased with age, with all individuals taken as the denominator. When the incidence of mumps deafness in our study is calculated based on all individuals surveyed, as in Fujiwara’s paper,^7^ the incidence of mumps deafness, contrary to that of sudden deafness, tends to decrease and becomes much lower with age. Therefore, we do not believe that the incidence of mumps deafness in our study is similar to that of sudden deafness.

Epidemiological studies that use a social insurance database may be inherently affected by the medical accuracy of individual diagnoses and age-related differences in pathology. Nonetheless, the analytic results of our study, which used the same definition for children and adults, still likely has some academic significance, despite the various study limitations, which is why we submitted our report. We hope that future studies will shed more light on this area.
